# Characteristics and health burden of the undiagnosed population at risk of chronic obstructive pulmonary disease in China

**DOI:** 10.1186/s12889-019-8071-8

**Published:** 2019-12-23

**Authors:** Marco Koch, Thomas Butt, Wudong Guo, Xue Li, Yirong Chen, Diana Tan, Gordon G. Liu

**Affiliations:** 1 Independent Research Consultant, Cologne, Germany; 2 UCL Institute of Ophthalmology, University College London, London, UK; 3 National School of Development, Peking University, Beijing, China; 4 China National Health Development Research Center, National Health Commission, Beijing, China; 5 Kantar Health, Singapore, Singapore; 6 Kantar Health, Shanghai, China

**Keywords:** Chronic obstructive pulmonary disease, China, National health and wellness survey, Health-related quality of life, Resource use, Productivity, Case detection, Screening, Lung function questionnaire

## Abstract

**Background:**

Chronic obstructive pulmonary disease (COPD) is a leading cause of morbidity and mortality in China. However, identifying patients has proved challenging, resulting in widespread under-diagnosis of the condition. We examined the prevalence of COPD diagnosis and COPD risk among adults in urban mainland China, the factors associated with having a COPD diagnosis or COPD risk, and the healthcare resource use and health outcomes of these groups compared with controls.

**Methods:**

Respondents to the 2017 National Health and Wellness Survey in China (*n* = 19,994) were classified into three groups: ‘COPD Diagnosed’, ‘COPD Risk (undiagnosed)’, and Control (unaffected), based on their self-reported diagnosis and Lung Function Questionnaire (LFQ) score. The groups were characterised by sociodemographic, health-related quality of life (HRQoL), productivity impairment, and healthcare resource use. Pairwise comparisons (t tests and chi-squared tests) and multivariable regression analyses were used to investigate factors associated with being at risk of, or diagnosed with, COPD.

**Results:**

3320 (16.6%) respondents had a suspected risk of COPD but did not report receiving a diagnosis. This was projected to 105.3 million people, or 16.9% of adult urban Chinese. Of these respondents with an identified risk, only 554 (16.7%) were aware of COPD by name. Relative to those without COPD, those with a risk of COPD (undiagnosed) had significantly greater healthcare resource use, lower productivity and lower HRQoL not only compared to those without COPD, but also compared to people with a COPD diagnosis. Factors associated with increased odds of being at risk of COPD were older age, smoking, alcohol consumption, overweight BMI, occasional exercise, higher comorbidities, asthma diagnosis, being female, lower education, not being employed, and living in a high pollution province (*p* < 0.05).

**Conclusions:**

There is a substantial group of individuals, undiagnosed, but living with a risk of COPD, who have impaired HRQoL, lower productivity and elevated healthcare resource use patterns. Case-detection tools such as the LFQ may prove a quick and cost-effective approach for identifying these at-risk individuals for further definitive testing and appropriate treatment in China.

## Background

Chronic Obstructive Pulmonary Disease (COPD) is characterised by progressive, partially irreversible airflow limitation [1]. The disease is one of the leading causes of death and disability globally, accounting for an estimated three million deaths per year and 2.6% of global disability-adjusted life years (DALYs) [2]. The burden of disease is most severe in low- and middle-income countries (LMICs) and people in these countries may have a different trajectory of disease due to exposure to different risk factors earlier in life [3, 4].

In China, COPD is the third leading cause of death and was responsible for over 0.9 million deaths in 2013 [5]. Several public health issues associated with COPD including pollution, smoking and an aging population are particularly acute in China [6]. The prevalence of spirometry-defined COPD was estimated to be 8.6% in adults 20 years and older by the China Pulmonary Health (CPH) study, equating to 99.9 million people [7]. However, COPD is underdiagnosed in China: only 2.6% of this spirometry-defined prevalent population were aware of their condition and 9.7% had a prior pulmonary function test [7]. Consequently, the overall burden of COPD in the community may be considerably underestimated.

The prognosis of diagnosed COPD patients can be improved through pharmacological and rehabilitative treatment and through the management of risk-factors [7]. Early diagnosis is important to achieve good outcomes. Forced expiratory volume_1_ (FEV_1_) declines at a faster rate in the early stages of the COPD and comorbidities are present from early in the disease [8, 9].

Although testing via spirometry is recommended for diagnosis of COPD, implementing a spirometry-based public screening programme in China faces considerable challenges including low awareness of the condition among the general population and non-specific symptoms [10]. Furthermore, spirometry is not widely available and would require a major investment and behavioural change in China’s large healthcare system [11].

Pre-screening questionnaires may offer a rapid, cost-effective approach to identify high-risk patients for screening spirometry and direct them for appropriate treatment and advice on minimising risk. The Lung Function Questionnaire (LFQ) is one such validated questionnaire, which has shown a sensitivity of 83–88% and specificity of 25–48% in international settings [12]. The LFQ has previously identified a large COPD risk group in Japan whom had poorer health outcomes and higher healthcare resource use compared with controls [13].

Since many people at risk of COPD are believed to reside in the community, and may never present in hospital, a representative general population survey containing a validated screening questionnaire offers an opportunity to investigate this population. To the best of our knowledge, this is the first study to characterise the diagnosed COPD population and those at-risk of COPD in China using a large, representative survey of the urban population. This study estimates the prevalence of adults diagnosed with COPD and those at risk for COPD (undiagnosed) based on the LFQ in urban China. It investigates the factors associated with having a COPD diagnosis or COPD risk and characterises the health outcomes and healthcare resource use of these groups.

## Methods

### Data source

The 2017 China National Health and Wellness Survey (NHWS) by Kantar Health is a cross-sectional, Internet-based survey self-administered in Chinese language to a nationwide sample of adults (aged 18 or older) who reside in an urban settlement. The sample (*n* = 19,994) is stratified by gender, age and region to represent the demographic composition of the urban adult population in China. Representativeness of the data has been validated and weighted against the official Chinese Statistical Yearbook [14]. Further methodologic details are provided in Additional file 1.

All respondents from the 2017 NHWS were included in the analysis and classified according to their self-reported physician diagnosis or at-risk status of COPD. In addition to the variables recorded in the NHWS, respondents were assigned an air pollution exposure status based on their province of residence, using provincial Air Quality Index (AQI) levels from the China Air Quality Online Monitoring and Analysis Platform.

### Measures

#### Diagnosis of COPD

To establish the diagnosis of a COPD-related condition, all respondents first indicated whether they ever had experienced “Chronic obstructive pulmonary disease (COPD)”, “Chronic bronchitis (cough up phlegm 4 of 7 days a week over last 3 months)” or “Emphysema”, among other diseases, and then whether their condition had been diagnosed by a physician. Respondents who self-reported a diagnosis of COPD, chronic bronchitis or emphysema were classified as ‘COPD Diagnosed’.

#### At-risk status of COPD

The LFQ is a five-item, disease-specific, self-report measure of COPD symptoms and risk factors [15]. It was developed as a convenient COPD pre-screening tool to determine in a primary-care setting which individuals may be at-risk for COPD and should therefore receive follow-up spirometry to confirm a diagnosis. The LFQ has been validated in COPD patients and has been previously used in several international settings [13, 16]. The LFQ was translated to Chinese by the NHWS study team.

Scores on the LFQ range from 5 (highest risk) to 25 (lowest risk). According to LFQ working group guidelines, respondents with LFQ scores between 5 and 18 may be considered at risk for COPD [16]. All respondents completed the LFQ. Those with a sum score of 18 or lower, but no self-reported COPD diagnosis, were classified as ‘COPD Risk (undiagnosed)’. Those with an LFQ sum score of 19 or greater, and no self-reported COPD diagnosis, were classified as ‘Control’. Those who reported a COPD diagnosis, regardless of risk score, were classified as ‘COPD Diagnosed’.

#### Awareness and subjective risk of COPD

All respondents were asked about their awareness of “Chronic Obstructive Pulmonary Disease (COPD)”, among a list of 22 conditions. Respondents also indicated which conditions out of a list they believed to be at risk for developing in the future. Those who selected any of the three COPD-related conditions (COPD, Chronic Bronchitis or Emphysema) were classified to have a “subjective risk of COPD”.

#### Demographics

Demographic variables for analysis included age, gender, marital status, education, employment status, monthly household income, and medical insurance type.

#### Health characteristics and comorbidities

Health history variables included smoking, alcohol consumption, exercise level, and body mass index (BMI). Under/overweight was defined by BMI levels of ≤18.5 and > 25 respectively, which reflect the international norms, although it should be noted that some have suggested different BMI levels be used for the Chinese population [17].

Health-related behaviours, such as presentation for an annual check-up with a healthcare provider (HCP) and usage of digital health & wellness tools (Apps, websites, wearables), were also assessed.

The burden from comorbidities was established using the Charlson comorbidity index (CCI) [18]. Higher total CCI scores indicate a greater burden. Respondents also reported whether they had a physician diagnosis of asthma. Since asthma is a commonly co-existing condition to COPD, [19, 20] and not part of the CCI, it was examined separately.

#### Health-related quality of life

HRQoL was assessed using the SF-12 and EQ-5D, which have been shown to be appropriate for use among people with COPD [21]. The SF-12 version 2 consists of twelve questions measuring eight health domains to assess physical and mental health. Physical (PCS) and mental (MCS) component summary scores can be derived [22]. The questionnaire and its component summary scores have been shown to be valid in the Chinese population [23].

The EQ-5D-5L is a standardised HRQoL instrument developed by the EuroQol Group. It comprises a descriptive system and a visual analogue scale (VAS) [24]. For analysis, the ratings of the five individual dimensions were combined to generate a single index utility value anchored by 0 (equivalent to dead) and 1 (full health) using the China value set.

Higher scores on PCS, MCS and EQ-5D indicate better health status. Differences in PCS and MCS scores exceeding 3 points, and differences in EQ-5D health utility exceeding 0.074 points, respectively, were considered minimally important differences (MID) from a clinical perspective [25].

#### Productivity impairment

Productivity impairment was assessed using the Work Productivity and Activity Impairment (WPAI) questionnaire [26]. Among those currently employed, the WPAI measured the effect of respondents’ general health on absenteeism (% of work time missed due to health problems), presenteeism (% of impairment while working) and overall work impairment (% of total combined work productivity loss) in the past seven days. Among all respondents, WPAI also assessed the perceived overall activity impairment (% of impairment in daily activities due to health problems in the past seven days). Higher values of the WPAI indicate greater impairment.

#### Healthcare resource use

Healthcare resource use was assessed by self-reported number of HCP visits (by specialty), emergency room (ER) visits and hospitalisations in the past six months for respondents’ “own medical condition”. The phrasing was intentionally not disease-specific, to capture total resource use due to any medical condition. The number of respiratory specialist HCP visits (pulmonologist, respiratory therapist and otolaryngologist visits) was calculated in addition to the total number of HCP visits.

#### Air pollution exposure

Average monthly provincial Air Quality Index (AQI) levels were taken from the China Air Quality Online Monitoring and Analysis Platform (www.aqistudy.cn) for the period 2014 to 2017 (48 months). The AQI is a summary measure of daily air quality widely used by national governments. In China, the total AQI score combines levels of six atmospheric pollutants: sulphur dioxide (SO_2_), nitrogen dioxide (NO_2_), suspended particulates smaller than 10 μm in aerodynamic diameter (PM_10_), suspended particulates smaller than 2.5 μm in aerodynamic diameter (PM_2.5_) carbon monoxide (CO), and ozone (O_3_) The AQI is calculated on a non-linear scale where a higher score indicates worse air pollution (greater danger to health).

We calculated the number of months with AQI levels of at least 101 (‘unhealthy for sensitive groups’). The five highest pollution provinces were Beijing (31 months AQI ≥ 101), Henan (27), Hebei (28), Tianjin (25), and Xinjiang (23). We also calculated average AQI levels between 2014 and 2017 and the same five provinces had the highest pollution levels: Hebei (average AQI 115), Beijing (114), Henan (112), Tianjin (108), and Xinjiang (104). Residents of these provinces were exposed to more ‘dangerous’ air months and higher average air pollution, both of which may contribute to COPD. Respondents residing in one of these provinces were classified as living in a high-pollution area.

### Statistical analysis

#### Descriptive and bivariate analyses

Prevalence estimates of a COPD diagnosis or an undiagnosed COPD risk status were calculated on the raw data and weighted data. The weighting procedure was performed in a multi-staged process accounting for age, gender and region (see Additional file 1). The weighted total sample was 621,668,160 adults in urban China, based on the Chinese Statistical Yearbook 2016 [14].

Descriptive statistics were used to examine the three groups (‘COPD Diagnosed’, ‘COPD Risk (undiagnosed)’ and ‘Control’) on demographics, health characteristics, and healthcare use. Those variables were also examined in bivariate comparisons between the three groups to test for differences, using independent-samples *t*-tests for continuous variables and chi-square tests for categorical variables. The results were the basis for identifying covariates for the multivariable models.

#### Multivariable analyses

Logistic regression was used to investigate demographic and health-related factors associated with a COPD diagnosis or an undiagnosed COPD risk status, respectively, compared to controls.

To assess the burden of COPD diagnosis or an undiagnosed COPD risk with respect to HRQoL, productivity impairment and healthcare resource use, generalized linear models specifying the appropriate distribution (normal for HRQoL variables, negative binomial for other variables) and link function (identity for HRQoL variables, log for other variables) were constructed. Adjusted means were calculated from the respective models for each outcome variable.

Statistical analyses were performed using R Version 3.5.2 (RStudio).

## Results

Out of 19,994 adults in NHWS, 513 (2.6%) self-reported a physician diagnosis of a COPD condition (among them 453 claiming a diagnosis with chronic bronchitis, 57 with emphysema, and 19 with COPD). 3534 (17.7%) respondents were identified with a suspected risk of COPD (measured by an LFQ score of 18 points or less). After excluding those with a self-reported diagnosis from the at-risk population, the ‘COPD Risk (undiagnosed)’ group equalled 3320 (16.6%).

Projecting these groups to the Chinese urban adult population of 622 million people, the diagnosed COPD population was estimated at 15.9 million (2.6%) and the undiagnosed COPD risk population at 105.3 million (16.9%).

Among all respondents, 12.4% (*n* = 2478) reported to be aware of COPD by name. Within the ‘COPD Diagnosed’, ‘COPD Risk (undiagnosed)’ and Control group, respectively, the awareness was at 21.8% (112), 16.7% (555) and 11.2% (1811). For comparison, awareness of the condition asthma in the three groups was at 66.5, 53.0 and 51.9%, respectively, and at 52.4% overall.

Only 3.9% (781) of all respondents felt to be at risk for developing a COPD condition in future ("subjective risk of COPD"). In the ‘COPD Risk (undiagnosed)’ group, 6.7% (222) felt to be at risk. Among the ‘COPD Diagnosed’ population, 55.4% (282) reported to be currently on a prescription medication for their COPD condition.

The ‘COPD Risk (undiagnosed)’ group was significantly more likely to be male, older, married, and state-health insured than controls, while likely being less educated, unemployed (retired: 44.6% versus 13.6%), and with a lower household income (Table 1). The ‘COPD Diagnosed’ population had a similar profile to the ‘COPD Risk (undiagnosed)’ group, but was generally less pronounced in their difference to the Control group, with marital status and income level differences being non-significant.

**Table 1:** Demographics of Control, ‘COPD Risk (undiagnosed)’ and ‘COPD Diagnosed’ groups

	All	Control group	‘COPD Risk (undiagnosed)’ group	‘COPD Diagnosed’ group	Risk vs. Control, *p* value	Diagnosed vs. Control, *p* value	Risk vs. Diagnosed, *p* value
	(*n* = 19,994)	(*n* = 16,161)	(*n* = 3320)	(*n* = 513)			
Gender, n (%)					< 0.001	< 0.001	< 0.001
Female	9994 (50.0)	8628 (53.4)	1135 (34.2)	231 (45.0)			
Male	10,000 (50.0)	7533 (46.6)	2185 (65.8)	282 (55.0)			
Age in years, n (%)					0.000	< 0.001	< 0.001
18–39	8369 (41.9)	7717 (47.8)	462 (13.9)	190 (37.0)			
40–59	7942 (39.7)	6491 (40.2)	1254 (37.8)	197 (38.4)			
60+	3683 (18.4)	1953 (12.1)	1604 (48.3)	126 (24.6)			
Marital status, n (%)					< 0.001	0.413	< 0.001
Married or living with partner	16,004 (80.0)	12,640 (78.2)	2951 (88.9)	413 (80.5)			
Not married	3954 (19.8)	3496 (21.6)	358 (10.8)	100 (19.5)			
Decline to answer	36 (0.18)	25 (0.15)	11 (0.33)	0 (0.00)			
Education, n (%)					< 0.001	< 0.001	< 0.001
Less than university degree	8312 (41.6)	5849 (36.2)	2200 (66.3)	263 (51.3)			
University (4 years) degree or higher	11,635 (58.2)	10,277 (63.6)	1109 (33.4)	249 (48.5)			
Decline to answer	47 (0.24)	35 (0.22)	11 (0.33)	1 (0.19)			
Household income (after tax/welfare), n (%)					< 0.001	0.880	< 0.001
CNY 7999 or below	13,606 (68.1)	10,718 (66.3)	2544 (76.6)	344 (67.1)			
CNY 8000–15,999	4809 (24.1)	4083 (25.3)	598 (18.0)	128 (25.0)			
CNY 16,000 or above	1321 (6.61)	1136 (7.03)	149 (4.49)	36 (7.02)			
Decline to answer	258 (1.29)	224 (1.39)	29 (0.87)	5 (0.97)			
Employment, n (%)					< 0.001	< 0.001	< 0.001
Not employed	5105 (25.5)	3276 (20.3)	1661 (50.0)	168 (32.7)			
Currently employed	14,889 (74.5)	12,885 (79.7)	1659 (50.0)	345 (67.3)			

Abbreviations: *CNY* Chinese Yuan (currency of the People’s Republic of China);

Both the ‘COPD Risk (undiagnosed)’ and ‘COPD Diagnosed’ groups were more likely to be smokers, drink more alcohol, exercise less, be more overweight, have more comorbidities (especially asthma), and attend annual health check-ups (Table 2). While the COPD Risk (undiagnosed) group less often used digital health tools, compared to controls, the COPD Diagnosed group used them more often.

**Table 2:** Health characteristics of Control, ‘COPD Risk (undiagnosed)’ and ‘COPD Diagnosed’ groups

	All	Control group	‘COPD Risk (undiagnosed)’ group	‘COPD Diagnosed’ group	Risk vs. Control, *p* value	Diagnosed vs. Control, *p* value	Risk vs. Diagnosed, *p* value
	(*n* = 19,994)	(*n* = 16,161)	(*n* = 3320)	(*n* = 513)			
Smoking, n (%)					0.000	< 0.001	< 0.001
Never smoked	15,019 (75.1)	13,342 (82.6)	1367 (41.2)	310 (60.4)			
Former smoker	1246 (6.23)	868 (5.37)	332 (10.0)	46 (8.97)			
Current smoker	3729 (18.7)	1951 (12.1)	1621 (48.8)	157 (30.6)			
Alcohol use, n (%)					< 0.001	< 0.001	0.030
2–3 times a week or more	3726 (18.6)	2542 (15.7)	1050 (31.6)	134 (26.1)			
Once a week or less often	7895 (39.5)	6327 (39.1)	1351 (40.7)	217 (42.3)			
I do not drink alcohol	8373 (41.9)	7292 (45.1)	919 (27.7)	162 (31.6)			
BMI, n (%)					< 0.001	0.008	0.023
Overweight/ Obese (BMI ≥ 25)	4272 (21.4)	3141 (19.4)	1006 (30.3)	125 (24.4)			
Normal (18.5 ≥ BMI < 25)	13,257 (66.3)	10,884 (67.3)	2032 (61.2)	341 (66.5)			
Underweight (BMI < 18.5)	1826 (9.13)	1595 (9.87)	194 (5.84)	37 (7.21)			
Decline to answer	639 (3.20)	541 (3.35)	88 (2.65)	10 (1.95)			
Exercise level (per month), n (%)					< 0.001	0.032	0.003
No exercise (0 days)	7272 (36.4)	5795 (35.9)	1281 (38.6)	196 (38.2)			
Occasional (1–10 days)	7388 (37.0)	6063 (37.5)	1118 (33.7)	207 (40.4)			
Frequent (11+ days)	5334 (26.7)	4303 (26.6)	921 (27.7)	110 (21.4)			
Charlson Comorbidity Index, mean ± SD					0.000	0.000	0.000
CCI	0.17 ± 0.52	0.10 ± 0.38	0.32 ± 0.74	1.37 ± 0.78			
Diagnosed of asthma, n (%)					< 0.001	< 0.001	< 0.001
Yes	357 (1.79)	191 (1.18)	117 (3.52)	49 (9.55)			
Annual physical check-up, n (%)					< 0.001	< 0.001	< 0.001
Yes	6451 (32.3)	4809 (29.8)	1378 (41.5)	264 (51.5)			
Digital health tools usage, n (%)					< 0.001	< 0.001	< 0.001
1+ tools used	8886 (44.4)	7337 (45.4)	1256 (37.8)	293 (57.1)			
None used	11,108 (55.6)	8824 (54.6)	2064 (62.2)	220 (42.9)			
COPD symptom scores (LFQ), mean ± SD							
Frequency of mucus production^a^	4.23 ± 0.83	4.43 ± 0.67	3.34 ± 0.87	3.57 ± 0.88	0.000	0.000	0.000
Frequency of wheezing^a^	4.55 ± 0.71	4.73 ± 0.50	3.74 ± 0.92	4.00 ± 0.86	0.000	0.000	0.000
Frequency of dyspnoea^a^	4.07 ± 0.91	4.27 ± 0.79	3.17 ± 0.86	3.58 ± 0.94	0.000	0.000	0.000
LFQ sum score, mean ± SD							
LFQ score	21.1 ± 3.00	22.2 ± 1.88	16.1 ± 1.89	18.8 ± 3.37	0.000	0.000	0.000

^a^ Scale 1 = very often to 5 = never

Abbreviations: *BMI* Body Mass Index; *COPD* Chronic Obstructive Pulmonary Disease; *LFQ* Lung Function Questionnaire; *SD* standard deviation

The three core symptoms of COPD captured in the LFQ - mucus production, wheezing and dyspnoea - were all significantly more frequent in the Diagnosed group than among controls, but even more frequent in the COPD Risk group (undiagnosed). The same pattern emerged regarding the LFQ sum score.

Both the ‘COPD Risk (undiagnosed)’ and ‘COPD Diagnosed’ groups showed significantly higher healthcare resource use, higher productivity impairment and worse HRQoL scores than the Control group (Table 3). Again, the gaps for most health outcome variables versus controls were more pronounced in the ‘COPD Risk’ population, compared to the ‘COPD Diagnosed’ population.

**Table 3:** Health outcomes of Control, ‘COPD Risk (undiagnosed)’ and ‘COPD Diagnosed’ groups

	All	Control group	‘COPD Risk (undiagnosed)’ group	‘COPD Diagnosed’ group	Risk vs. Control, *p* value	Diagnosed vs. Control, *p* value	Risk vs. Diagnosed, *p* value
	(*n* = 19,994)	(*n* = 16,161)	(*n* = 3320)	(*n* = 513)			
Healthcare resource use (in past 6 months), mean ± SD							
ER visits	0.32 ± 1.00	0.26 ± 0.87	0.53 ± 1.38	0.68 ± 1.42	0.000	0.000	0.004
Hospitalisations	0.13 ± 0.83	0.09 ± 0.81	0.31 ± 0.91	0.27 ± 0.74	0.000	< 0.001	0.534
HCP visits	1.49 ± 3.53	1.30 ± 3.39	2.12 ± 3.86	3.28 ± 4.55	0.000	0.000	0.000
Respiratory specialist visits	0.08 ± 0.69	0.07 ± 0.56	0.11 ± 1.11	0.39 ± 0.93	0.003	0.000	0.000
WPAI, mean ± SD							
%Absenteeism^a^	3.93 ± 10.8	3.22 ± 9.62	8.87 ± 16.2	6.75 ± 13.8	< 0.001	< 0.001	0.002
%Presenteeism^a^	21.5 ± 24.6	19.7 ± 23.9	34.0 ± 27.1	26.4 ± 22.7	< 0.001	< 0.001	< 0.001
%Overall work impairment^a^	23.4 ± 26.0	21.4 ± 25.0	37.7 ± 28.9	29.7 ± 25.1	< 0.001	< 0.001	< 0.001
%Activity impairment	20.6 ± 23.1	18.2 ± 22.0	31.1 ± 25.0	27.0 ± 23.1	0.000	0.000	< 0.001
HRQoL, mean ± SD							
MCS (SF-12v2)	48.5 ± 7.92	49.0 ± 7.82	46.6 ± 7.99	45.6 ± 7.89	0.000	0.000	0.029
PCS (SF-12v2)	50.7 ± 6.83	51.7 ± 6.28	46.3 ± 7.44	47.5 ± 6.96	0.000	0.000	< 0.001
Health Utility (EQ-5D)	0.93 ± 0.12	0.94 ± 0.10	0.87 ± 0.18	0.88 ± 0.15	0.000	0.000	0.067

^a^ Absenteeism, Presenteeism and Overall Work Impairment calculated for the employed population only (*n* = 14,889)

Abbreviations: *ER* Emergency Room; *HCP* Healthcare provider; *HRQoL* Health-related quality of life; *SF-12v2* Short Form-12 version 2; *MCS* mental component summary; *PCS* physical component summary; *SD* Standard Deviation; *WPAI* Work Productivity and Activity Impairment

**Fig. 1:**
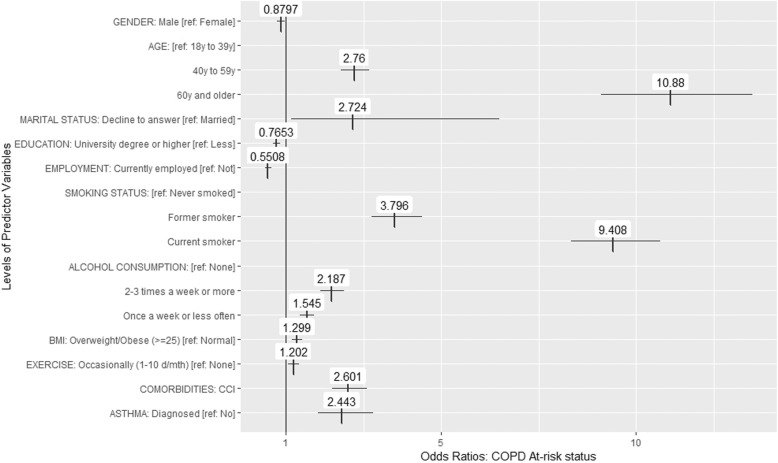
Logistic regression showing odds of being part of the ‘COPD Risk (undiagnosed)’ group (vs. Control group) as a function of predictors, adjusting for covariates. All predictors shown in the graphic reached statistical significance at *p* < 0.05. Tested predictors that did not reach significance include: Not married [ref. Married/living with partner]; Education level Decline to answer [ref. Less than university degree]; Monthly household income after deducting employer welfare benefits: All levels [ref. CNY 7999 or less]; BMI Underweight [ref. BMI Normal]; BMI Decline to answer [ref. BMI Normal]; Frequent exercise (11+ days/month) [ref. No exercise]

Figure 1 shows factors that are associated with being at an undiagnosed risk of COPD. Logistic regression models showed that older age (40–59 & 60+ vs. 18–39), smoking, alcohol consumption, overweight BMI level, occasional exercise (1–10 days per month vs. no exercise), higher CCI score, diagnosis of asthma, being female, having less than a university degree, and not being employed were all significantly, independently associated with being at an undiagnosed risk of COPD, relative to controls.

Table 4 shows the results of regression models investigating the association of ‘COPD Risk’ status with HRQoL, healthcare resource use, and productivity impairment, controlled for the independent predictors of ‘COPD Risk’ status (details: see table footnote).

**Table 4:** Health-related quality of life, healthcare resource use and productivity outcome measures as a function of ‘COPD Risk (undiagnosed)’ status

Outcome	Group	Coefficient (β/ IRR)	P value	Adjusted mean	SE
MCS (SF-12v2)	Controls (ref.)			47.44	0.59
(*n* = 19,481)	COPD Risk	β: − 3.75	< 0.000	43.70	0.59
PCS (SF-12v2)	Controls (ref.)			47.01	0.49
(*n* = 19,481)	COPD Risk	β: −4.28	< 0.000	42.73	0.50
Health utility (EQ-5D)	Controls (ref.)			0.86	0.01
(*n* = 19,481)	COPD Risk	β: −0.08	< 0.000	0.61	0.01
ER visits	Controls (ref.)			0.41	0.03
(*n* = 18,616)	COPD Risk	IRR: 1.71	< 0.000	0.70	0.06
Hospitalisations	Controls (ref.)			0.11	0.01
(*n* = 18,616)	COPD Risk	IRR: 2.92	< 0.000	0.31	0.04
HCP visits	Controls (ref.)			2.16	0.11
(*n* = 18,616)	COPD Risk	IRR: 1.31	< 0.000	2.83	0.15
Respiratory specialist visits	Controls (ref.)			0.17	0.03
(*n* = 18,616)	COPD Risk	IRR: 1.38	0.008	0.23	0.04
Absenteeism	Controls (ref.)			4.26	0.58
(*n* = 13,658)	COPD Risk	IRR: 2.46	< 0.000	10.48	1.55
Presenteeism	Controls (ref.)			21.57	1.52
(*n* = 13,658)	COPD Risk	IRR: 1.74	< 0.000	37.51	2.85
Overall work impairment	Controls (ref.)			24.10	1.66
(*n* = 13,658)	COPD Risk	IRR: 1.74	< 0.000	42.01	3.13
Activity impairment	Controls (ref.)			20.85	1.12
(*n* = 18,616)	COPD Risk	IRR: 1.71	< 0.000	35.69	2.02

Note: All models adjusted for gender, age, marital status, education level, household income, employment status, smoking status, alcohol consumption level, body mass index, exercise level, Charlson Comorbidity Index and self-reported asthma diagnosis. Betas (β) were from normal regression models, whereas Incident Rate Ratios (IRR) were from negative binomial models. Models predicting Absenteeism, Presenteeism and Overall Work Impairment are limited to the employed population, while employment status has been dropped as a control variable in these models accordingly. Abbreviations: *SF-12v2* Short Form-12 version 2; *MCS* mental component summary; *PCS* physical component summary; *ER* emergency room; *HCP* healthcare provider; *SE* standard error

Relative to controls, COPD risk was a significant predictor of poorer HRQoL in all models. COPD risk was associated with lower MCS, lower PCS, and lower EQ-5D health utility score.

Relative to controls, COPD risk was a significant independent predictor of higher healthcare resource use in the past six months, namely a higher frequency of ER visits, hospitalisations, and healthcare provider visits, with a higher frequency of respiratory specialist visits in particular.

Relative to controls, COPD risk was a significant predictor of lower productivity, with higher levels of absenteeism, presenteeism, overall work impairment, and activity impairment.

We also investigated the association of ‘COPD Diagnosed’ status with health outcomes (see Additional file 2). Relative to controls, COPD diagnosis was a significant predictor of lower MCS and EQ-5D scores, as well as a higher frequency of respiratory specialist visits. However, no significant associations were found for other resource use variables, nor for the productivity measures or the PCS.

Given the high levels of air pollution exposure faced by many urban Chinese residents, we additionally investigated the impact of living in a high pollution area on ‘COPD Risk’ status. 3520 (17.6%) respondents lived in one of the five provinces defined as ‘high-pollution areas’. Pairwise comparisons exhibited no significant differences in COPD status versus the Control group (‘COPD Risk (undiagnosed)’ vs. Control, *p* = 0.192; ‘COPD Diagnosed’ vs. Control, *p* = 0.154). However, after controlling for covariates, a logistic regression model showed that living in a province with high AQI levels was a significant independent predictor of being at an undiagnosed risk for COPD (OR 1.203, *p* = 0.002). (see Additional file 3 for details).

## Discussion

This study found a large population at risk of COPD. We estimated an undiagnosed COPD risk population of 105.3 million adult urban residents. While we cannot reliably project this to the overall Chinese population due to different risk factors between rural and urban areas, given that urban residents make up just slightly more than half of the Chinese population (56.1% vs. 43.9% rural), and other studies have found higher rates of COPD in rural areas, [28] there may be a similar or greater number of people at risk of COPD living in rural China.

The ‘COPD Diagnosed’ group in our study was small, with only 2.6% reporting a COPD diagnosis from a physician. This prevalence rate is lower than other studies in clinical settings that calculated prevalence rates based on spirometer testing of patients. A systematic review reported the prevalence rate of COPD in China to be 5.87% [28]. We believe the difference is due to our study being conducted among the general population and therefore relying on people having received prior clinical testing for COPD. Studies in clinical settings have typically found low levels of prior diagnosis. In the CPH study, among participants with spirometry-defined COPD only 2.6% were aware of their condition [7].

The finding is consistent with other studies indicating that many patients who were found to have COPD via spirometry had not previously been diagnosed with COPD [7]. It is also consistent with our findings that there is a lack of awareness of the condition among the general population and the population at risk of COPD. Among the diagnosed population, awareness of the term “COPD” was also low, indicating that patients know COPD-related disease by a range of different terms.

Our findings that a range of sociodemographic factors are predictive of COPD are consistent with previous research that showed education and household income were risk factors for self-reported COPD in China [29]. It may allow for targeted screening to be undertaken to identify high risk individuals. For example, the finding that significantly more at-risk people reported undertaking annual health check-ups means that health centres performing these examinations may be a fruitful place to conduct screening programmes for COPD patients.

We identified some different features between the ‘COPD Diagnosed and ‘COPD Risk (undiagnosed)’ populations. Firstly, the overlap between the two groups was low, with 299 (58.3%) of ‘COPD Diagnosed’ patients being classified as ‘not-at-risk’. This may indicate that being diagnosed with COPD facilitates disease management and avoidance of risk factors that mean that the condition is better controlled and therefore the LFQ score is lowered. Supporting this hypothesis, we found that being at risk of COPD was a significant predictor of diminished patient outcomes and increased healthcare resource use and that these signals were stronger in the risk group than in the diagnosed group.

In the absence of widespread systematic spirometry screening for COPD and its risk factors in China, there is interest in approaches to raise disease awareness and diagnosis [10]. From a humanistic standpoint, while COPD prevalence is half that of asthma, it is considerably more deadly, accounting for eight times more deaths globally [2]. From a healthcare system perspective, estimates of direct medical costs between USD72 to USD3,565 per patient per year (CNY507 to CNY25,100; USD1.00:CNY7.04) [28]. Costs incurred during an acute exacerbation are about 40–70% of overall treatment costs while the average length of an inpatient hospital stay for each acute exacerbation episode was reported to be 20.7 days, with average costs of CNY22,691 (USD3,223; USD1.00:CNY7.04) [30].

There are several reasons that identification of COPD patients has proved challenging. Firstly, awareness levels among clinicians and patients are generally low. Many local physicians are not familiar with specific symptoms of COPD, and they typically mistake COPD as bronchiolitis or pneumococcal pneumonia [30]. There are significant regional variations in disease awareness, diagnosis and outcomes. One study found a tenfold difference in prevalence of airflow obstruction between different regions due to different risk factors and high levels of misdiagnosis [27].

In rural areas, patients may be lacking basic knowledge of COPD, its risk factors and treatment options. Among 8217 patients in a rural area of China who were identified as having COPD from their medical records, 96% had not heard of COPD when they were interviewed, while almost one third did not realise that smoking was a risk factor [31]. Introducing a standardised questionnaire such as the LFQ into the healthcare system may help improve case detection and reduce rates of misdiagnosis, particularly in resource- or knowledge-poor settings.

Secondly, perverse financial incentives have driven historic over- and under-use of diagnostics and treatments. Studies have found high use of antibiotics and corticosteroids in China including a level of antibiotic prescription twice the level recommended by the World Health Organization (WHO) [32, 33]. Among the 8217 COPD patients in rural China, none used common management options such as inhalers, nebuliser drugs and oxygen therapy. Instead, 51% took theophylline to relieve dyspnoea, and 42% used antibiotics to treat exacerbations [31]. Identifying signs of COPD and intervening early may help to manage patients in a better state of health reducing the need to resort to antibiotics and corticosteroids.

This study suggests that pre-screening tools such as the LFQ may provide a quick and cost-effective approach for identifying individuals at risk of COPD for further definitive testing. In parallel with measures to improve identification of patients, a program of prevention via pneumococcal vaccination, improving and standardising treatment is needed over the longer term to reduce regional disparities in treatment patterns and outcomes, such as that undertaken by the China National Health Development Research Center (CNHDRC) to reform the COPD clinical pathway in rural areas [30].

The findings of this study such as low awareness, underdiagnosis, and particular risk factors are common in many Asian countries, therefore this study may be generalisable to other countries in the Asia region [34]. There may be other specific issues related to COPD in Asian populations such as the relatively low BMI of patients, that means COPD studies may not be generalisable more widely.

Our study has several limitations. Outcomes were self-reported and not clinically verified. As such, the findings may be limited by recall issues. Further details on COPD-specific symptomatology are contained within the NHWS, however since these were only collected from those with a self-reported diagnosis, it was not possible to use these to further characterise the population at risk of COPD. Additionally, this was a cross-sectional study, and results may thus not represent how the relationships of interest change over time and neither can causal relationships be established. While the NHWS is broadly representative of the Chinese adult urban population, its representativeness of the diagnosed and at-risk COPD subpopulations is unknown. It is possible that low estimates of COPD diagnosis may reflect a bias in the study design towards relatively younger, healthier and/or wealthier respondents, who have easier access to online studies.

Previous studies have attributed airborne PM2.5 to increased mortality from respiratory diseases including COPD [6]. The link between risk of COPD and air pollution is highly relevant to Chinese policymakers as urban Chinese residents are exposed to high levels of airborne pollutants. In this study we identified air pollution as an independent factor that contributes to COPD at-risk status. However, risk is likely to be a non-linear function including multiple sources of PM2.5 (principally air, tobacco and solid fuel), other particles such as PM10, exposure levels and historic exposure, [35, 36] therefore we would recommend further work to understand this association.

The LFQ is a well validated case detection tool for COPD. However, it was originally developed in the USA and therefore it may benefit from validation and/or adaptation to the Chinese context. For example, it may be appropriate to incorporate some risk factors that are more prevalent in China such as exposure to indoor air pollution from heating and cooking with solid fuel.

## Conclusions

This study found a substantial population suspected to be at risk of COPD, but undiagnosed, who have impaired HRQoL and elevated healthcare resource use. The LFQ or an adapted version may prove a quick and cost-effective approach for identifying these at-risk individuals for further definitive testing.

## Supplementary information

**Additional file 1.** Further details on study methodology. Additional information on study methodology including study recruitment, covariates, the Lung Function Questionnaire, and sample weighting.

**Additional file 2.** Health-related quality of life, healthcare resource use and productivity outcome measures as a function of ‘COPD Diagnosed’ status. Table showing logistic regression for the ‘COPD Diagnosed’ group.

**Additional file 3.** Supplemental analyses on the impact of provincial air pollution exposure on COPD status. Table and figure showing the results of group comparisons by air pollution exposure status and an odds ratio model where pollution exposure status was added to the list of predictors.

## Data Availability

The data that support the findings of this study are available from Kantar Health but restrictions apply to the availability of these data, which were used under license for the current study, and so are not publicly available. Data are however available from the authors upon reasonable request and with permission of Kantar Health.

## Abbreviations

AQI: Air quality index
BMI: Body mass index
CCI: Charlson comorbidity index
CNY: Chinese yuan
COPD: Chronic obstructive pulmonary disease
DALY: Disability-adjusted life year
ER: Emergency room
FEV: Forced expiratory volume
HCP: Health care provider
HRQoL: Health-related quality of life
LFQ: Lung Function Questionnaire
LMIC: Low- and middle-income countries
NHWS: National Health and Wellness Survey
VAS: Visual analogue scale
WHO: World Health Organization
WPAI: Work Productivity and Activity Impairment
