# Supplementary material for: Characteristics and health burden of the undiagnosed population at risk of chronic obstructive pulmonary disease in China
Source: BMC Public Health. 2019 Dec 23;19:1727. doi: 10.1186/s12889-019-8071-8 (PMC6929419; doi:10.1186/s12889-019-8071-8)
Supplement: Supplementary file 1 — Additional file 1. Further details on study methodology. Additional information on study methodology including study recruitment, covariates, the Lung Function Questionnaire, and sample weighting. [file 12889_2019_8071_MOESM1_ESM.docx]

**Additional file 1: Further details on study methodology**

**Study Recruitment**

Most respondents of the 2017 China NHWS (n=15,960 or 79.8%) were recruited via Internet panels. They accessed the survey through an email invitation received from their panel provider and completed it using a web-based format.

Respondents were invited to the study based on their place of residence and the survey included a question on the city of residence to confirm that respondents live in an urban settlement as defined by the National Bureau of Statistics of China (<http://www.stats.gov.cn/enGliSH/ClassificationsMethods/Definitions/200204/t20020424_72391.html>).

To ensure adequate representation of the elderly and those without proper Internet access, computer-assisted web-interviews (CAWI) were completed by a total of 4,034 respondents (20.2%) aged 50 years and older in ten of the largest urban regions: Beijing, Shenyang, Xi’an, Hefei, Changsha, Chengdu, Shanghai, Jinan, Guiyang, and Guangzhou. The CAWI respondents were recruited offline, invited to a centralized facility and asked to complete the survey on a web-connected computer with the help of an interviewer, as needed.

The exclusion criteria for the survey are:

• Respondents aged under 18 years old

• Declined to provide informed consent

• Respondents unable to complete survey on their own (not comfortable with a computer, poor eyesight, pain in hands/wrist, other) and declined to participate with the assistance of an interviewer.

All NHWS respondents were compensated for their participation with nominal incentives. The survey response rate was 78%.

**Covariate Measures**

The patient characteristics that were identified as being statistically significant in bivariate group comparisons and chosen as covariates in the multivariable regression models include the following categorical and continuous variables:

Demographics: Age (grouped 18-39 years; 40-59 years; 60 years and older), sex (male vs. female), marital status (married/living with partner vs. single/divorced/separated/widowed), education (university degree (4 years) or higher vs. less), employment status (full-time/part-time/self-employed vs. retired/student/unemployed), monthly household income after deducting employer-paid social welfare benefits (RMB 7,999 or below; RMB 8,000 to 15,999; RMB 16,000 or above), and medical insurance type (state insurance; commercial or other insurance; no insurance). Health characteristics: Smoking (current smoker; former smoker; never smoked), alcohol consumption (2-3 times per week or more; once a week or less; drink no alcohol), days of physical exercise in past month (frequent (11+ days); occasional (1-10 days) vs. no exercise) body mass index (BMI score), Charlson Comorbidity Index (CCI score*), self-reported Asthma diagnosis (yes vs. no).

* The CCI score is calculated as a weighted sum of the following conditions: HIV/AIDS, metastatic tumour, lymphoma, leukaemia, any tumour, moderate/severe renal disease, hemiplegia, diabetes, mild liver disease, ulcer disease, connective tissue disease, chronic pulmonary disease, dementia, cerebrovascular disease, peripheral vascular disease, myocardial infarction, congestive heart failure, and diabetes with end organ damage.

**Lung Function Questionnaire (LFQ)**

The LFQ is a five-item, disease-specific, self-report measure of COPD symptoms and risk factors. It includes experienced frequency of mucus production (“How often do you cough up mucus?”), wheezing (“How often does your chest sound noisy (wheezy, whistling, rattling) when you breathe?”) and dyspnoea (“How often do you experience shortness of breath during physical activity (walking up a flight of stairs or walking up an incline without stopping to rest)?”), with response options ranging from 1 = “very often” to 5 = “never”. In addition, it captures respondents’ smoking history (“How many years have you smoked?” with response options 1 = ”more than 30 years” to 5 = “never smoked”) and age group (“What is your age” with response options 1 = “70 years or older” to 5 = “less than 40 years”).

Since the LFQ records specific COPD symptoms, data from all respondents was available regarding their experienced frequency of mucus production, wheezing and dyspnoea, with response options from 1 = “very often” to 5 = “never”. The symptom scores and the total LFQ score were analysed. Lower values indicate greater symptoms.

Further information on COPD characteristics was captured from the diagnosed population, including whether they currently use a prescription medication to treat their COPD.

**Sample Weighting**

The NHWS used a stratified-random sampling framework in which the proportions of the adult Chinese urban population were matched on age and gender based on the Chinese statistical yearbook. The weighting procedure was performed in a multi-staged process: First, the age, gender, and region distribution for the NHWS study sample was computed. Then, the related distribution for the adult urban population was retrieved from the Chinese Statistical Yearbook 2016. Sampling weights were calculated using a raking procedure to ensure representativeness with respect to age, gender, and urban area. Eventually, these weights were assigned to reach respondent based on their cell membership (i.e., which age group, gender, and region they belonged to) and used for computing the relevant analyses.
