# Supplementary material for: Characteristics and health burden of the undiagnosed population at risk of chronic obstructive pulmonary disease in China
Source: BMC Public Health. 2019 Dec 23;19:1727. doi: 10.1186/s12889-019-8071-8 (PMC6929419; doi:10.1186/s12889-019-8071-8)
Supplement: Supplementary file 2 — Additional file 2. Health-related quality of life, healthcare resource use and productivity outcome measures as a function of ‘COPD Diagnosed’ status. Table showing logistic regression for the ‘COPD Diagnosed’ group. [file 12889_2019_8071_MOESM2_ESM.docx]

**Additional file 2: Table on health-related quality of life, healthcare resource use and productivity outcome measures as a function of COPD diagnosed status**

| Outcome | Group | Coefficient (β/ IRR) | P value | Adjusted mean | SE |
| --- | --- | --- | --- | --- | --- |
| MCS (SF-12v2) | Controls (ref.) |  |  | 46.83 | 0.67 |
| (n=19,481) | COPD Diagnosed | β: -1.20 | 0.002 | 45.63 | 0.76 |
| PCS (SF-12v2) | Controls (ref.) |  |  | 47.00 | 0.55 |
| (n=19,481) | COPD Diagnosed | β: -0.56 | 0.075 | 46.43 | 0.62 |
| Health utility (EQ-5D) | Controls (ref.) |  |  | 0.88 | 0.01 |
| (n=19,481) | COPD Diagnosed | β: -0.01 | 0.004 | 0.86 | 0.01 |
|  | | | | | |
| ER visits | Controls (ref.) |  |  | 0.43 | 0.04 |
| (n=18,616) | COPD Diagnosed | IRR: 1.13 | 0.297 | 0.49 | 0.07 |
| Hospitalisations | Controls (ref.) |  |  | 0.13 | 0.02 |
| (n=18,616) | COPD Diagnosed | IRR: 1.06 | 0.749 | 0.13 | 0.03 |
| HCP visits | Controls (ref.) |  |  | 2.27 | 0.12 |
| (n=18,616) | COPD Diagnosed | IRR: 1.01 | 0.842 | 2.30 | 0.19 |
| Respiratory specialist visits | Controls (ref.) |  |  | 0.13 | 0.02 |
| (n=18,616) | COPD Diagnosed | IRR: 4.64 | <0.000 | 0.61 | 0.14 |
|  | | | | | |
| Absenteeism | Controls (ref.) |  |  | 4.92 | 0.76 |
| (n=13,658) | COPD Diagnosed | IRR: 0.98 | 0.835 | 4.71 | 1.14 |
| Presenteeism | Controls (ref.) |  |  | 22.77 | 1.79 |
| (n=13,658) | COPD Diagnosed | IRR: 1.74 | 0.867 | 22.38 | 2.74 |
| Overall work impairment | Controls (ref.) |  |  | 25.63 | 1.98 |
| (n=13,658) | COPD Diagnosed | IRR: 1.00 | 0.972 | 25.54 | 3.06 |
| Activity impairment | Controls (ref.) |  |  | 22.57 | 1.43 |
| (n=18,616) | COPD Diagnosed | IRR: 1.04 | 0.618 | 23.56 | 2.34 |

Note: All models adjusted for gender, age, marital status, education level, household income, employment status, smoking status, alcohol consumption level, body mass index, exercise level, Charlson Comorbidity Index, and self-reported asthma diagnosis. Betas (β) were from normal regression models, whereas Incident Rate Ratios (IRR) were from negative binomial models. Models predicting Absenteeism, Presenteeism, and Overall Work Impairment are limited to the employed population, while employment status has been dropped as a control variable in these models accordingly. Abbreviations: SF-12v2, Short Form-12 version 2; MCS, mental component summary; PCS, physical component summary; ER, emergency room; HCP, healthcare provider; SE, standard error.
