# Supplementary material for: Characteristics and health burden of the undiagnosed population at risk of chronic obstructive pulmonary disease in China
Source: BMC Public Health. 2019 Dec 23;19:1727. doi: 10.1186/s12889-019-8071-8 (PMC6929419; doi:10.1186/s12889-019-8071-8)
Supplement: Supplementary file 3 — Additional file 3. Supplemental analyses on the impact of provincial air pollution exposure on COPD status. Table and figure showing the results of group comparisons by air pollution exposure status and an odds ratio model where pollution exposure status was added to the list of predictors. [file 12889_2019_8071_MOESM3_ESM.docx]

**Additional file 3: Supplemental analyses on the impact of provincial air pollution exposure on COPD status**

**Table 3-1: Air pollution exposure status, compared among Control, COPD Risk (undiagnosed) and COPD Diagnosed groups.**

|  | All | Control group | COPD Risk group (undiagnosed) | COPD Diagnosed group | Risk vs. Control, p value | Diagnosed vs. Control, p value | Risk vs. Diagnosed, p value |
| --- | --- | --- | --- | --- | --- | --- | --- |
|  | (n=19,994) | (n=16,161) | (n=3,320) | (n=513) |  |  |  |
| Living in a ‘high-pollution’ province, n (%) | | | | | 0.192 | 0.154 | 0.192 |
| Yes | 520  (17.6%) | 2,887  (17.9%) | 559  (16.8%) | 74  (14.4%) |  |  |  |
| No | 6,474  (82.4%) | 13,274  (82.1%) | 2,761  (83.2%) | 439  (85.6%) |  |  |  |

Note: Based on the average monthly provincial Air Quality Index (AQI) levels for the period 2014 to 2017 (48 months), respondents were classified as “living in a ‘high-pollution’ province” if they resided in one of the following Chinese provinces: Hebei, Beijing, Henan, Tianjin, and Xinjiang (Details: see Methods section of main manuscript).

**Figure 3-1: Logistic regression showing odds of being at risk for COPD (vs. control group) as a function of predictors, adjusting for covariates including “Living in a ‘high-pollution’ province”**


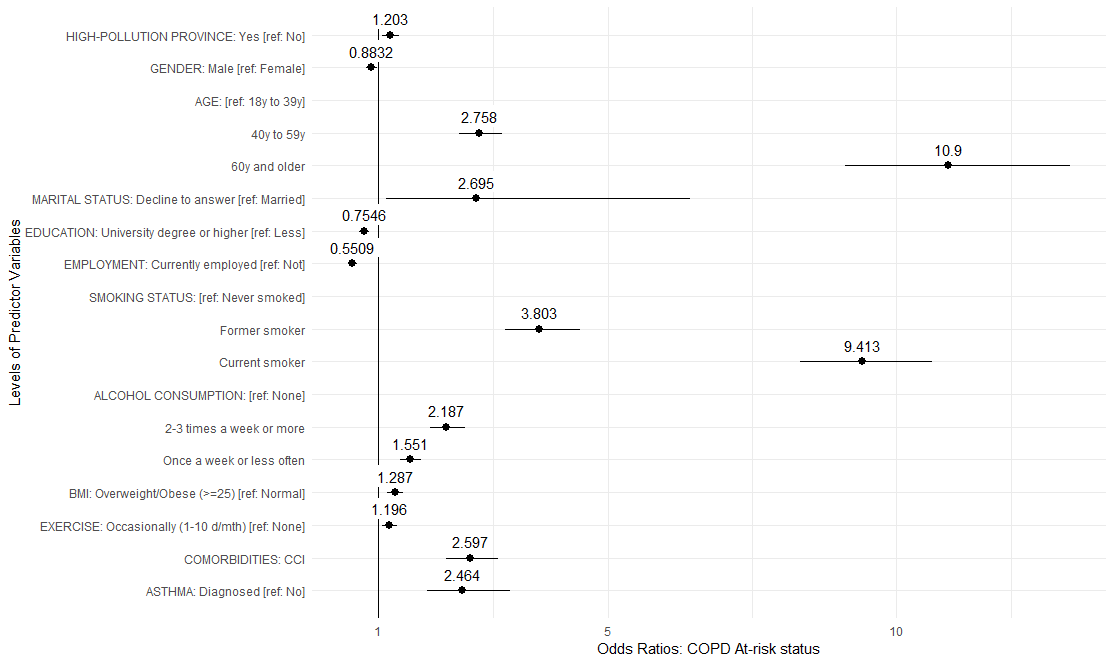


All predictors shown in the graphic reached statistical significance at p<0.05. Tested predictors that did not reach significance include: Not married [ref. Married/living with partner]; Education level Decline to answer [ref. Less than university degree]; Monthly household income after deducting employer welfare benefits: All levels [ref. CNY 7,999 or less]; BMI Underweight [ref. BMI Normal]; BMI Decline to answer [ref. BMI Normal]; Frequent exercise (11+ days/month) [ref. No exercise].
